# Renewed Feedback-Informed Group Treatment for Patients with Anxiety and Depressive Disorders

**DOI:** 10.1007/s10488-023-01338-y

**Published:** 2024-01-30

**Authors:** Marjolein M. W. Koementas-de Vos, Bea Tiemens, Fabiana Engelsbel, Kim de Jong, Cilia L. M. Witteman, M. Annet Nugter

**Affiliations:** 1 GGZ Noord-Holland-Noord, Heerhugowaard, The Netherlands; 2 Behavioural Science Institute, Radboud University, Nijmegen, The Netherlands; 3 Pro Persona Research, Nijmegen, The Netherlands; 4 Institute of Psychology, Clinical Psychology Unit, Leiden University, Leiden, The Netherlands

**Keywords:** Feedback, Group psychotherapy, Coaching, Group therapeutic factors

## Abstract

Feedback-Informed Group Treatment (FIGT) shows promise for improving outcomes, but results are mixed. The aim was investigating the feasibility, acceptability and effects of renewed FIGT on clinical outcomes and therapy processes. In a quasi-experimental pilot study, 65 patients with anxiety or depressive disorders and 15 therapists of interpersonal psychotherapy or cognitive behavioural therapy groups using renewed FIGT were included. Renewed FIGT contained three additions compared to the previous tool: (1) personalized goals along with the Outcome Questionnaire-45 (OQ-45), (2) therapists’ training, coaching and intervision, and (3) instructions to actively use feedback in the group. Data on feasibility, acceptability, outcomes and process factors were analysed and compared with those of historical cohorts using only OQ-45 feedback or no feedback, using descriptive, multilevel and covariance statistical analyses. Feasibility was mostly improved, with patients experiencing more feedback discussions and better usability compared to only OQ-45 feedback. At least two thirds of the patients and therapists give preference to using feedback in the future. At the end of the study, therapists were less convinced that the OQ-45 and goals were able to detect change. Renewed FIGT did not improve effectiveness on clinical outcomes. Compared to no feedback, patients experienced more cohesion, engagement and less avoidance, but improved less on depressive symptoms. Even when renewed FIGT is more feasible and usable than only OQ-45 feedback and associated with more cohesiveness and engagement, it may not automatically lead to improved effectiveness on clinical outcomes in short-term group therapy. Implications and future directions are described.

## Introduction

### Psychotherapy Is Not Optimal for Every Patient

Group psychotherapy appears to be equally effective in the treatment of mental disorders as individual psychotherapy (Burlingame et al., 2016). Despite its comparable effectiveness, group psychotherapy is a unique form of treatment that enables patients to empathize with, learn from and help other group members (Fuhriman & Burlingame, 1990). As with individual therapy, not all patients appear to benefit as expected. It is estimated that 15–25% drop-out prematurely and 5–15% of patients deteriorate at the end of treatment (Barkowski et al., 2020; Fernandez et al., 2015; Hans & Hiller, 2013; Slone et al., 2015). Unfortunately, therapists are poor in detecting these negative changes during treatment (Chapman et al., 2012; Hatfield et al., 2010; Walfish et al., 2012).

### Feedback-Informed Treatment (FIT)

Feedback-Informed Treatment (FIT), or so-called measurement based care or routine outcome monitoring, has been developed to monitor treatment progress by the use of self-report questionnaires, aiming to detect stagnation or deterioration in time, feeding back results to the patient and / or therapists, and allowing adjustments to the treatment if needed (Lambert, 2017; Lutz et al., 2015). FIT has been studied primarily in individual therapeutic settings and it appears to be particularly effective for patients who benefit less from treatment than expected, also known as not-on-track patients (NOT; Lambert, 2017). More recently it has been found that feedback is effective for all patients in improving symptoms and reducing dropout rates by 20% (De Jong et al., 2021) and that feedback is a cost-effective strategy for improving outcomes (Delgadillo et al., 2021). Despite these promising results, the implementation of FIT appears to be challenging (Lewis et al., 2019) and there is evidence that the effect disappears when implementation is poor (Bickman et al., 2016). It has been found that therapists are more negative about the use of feedback than patients and experience feedback as time-consuming and complicated. Patients mainly see benefits in visualizing their therapy progress and discussing it with their therapist, but may also have concerns, especially if the purpose is unclear or the feedback is too limited in describing their experiences and needs (Callaly et al., 2006; Moltu et al., 2018; Solstad et al., 2019; Thew et al., 2015; Unsworth et al., 2012).

### Feedback-Informed Group Treatment (FIGT)

Less is known about the effects of feedback in group psychotherapy. So far, eight studies of feedback-informed group treatment (FIGT) have been published and the results are mixed. Four FIGT studies demonstrated a positive effect of FIGT for both NOT-patients and on-track (OT) patients (Hutson et al., 2020; Koementas-de Vos et al., 2018; Schuman et al., 2015; Slone et al., 2015), two studies described benefits for NOT patients (Burlingame et al., 2018b; Newnham et al., 2010), one study found no effect at all (Davidsen et al., 2017) and one study found negative effects on effectiveness on depressive symptoms and quality of life (Koementas-de Vos et al., 2022b). The results on attendance also appear to vary between the FIGT studies. Two studies showed that patients with FIGT attended more therapy sessions and showed greater improvement in symptoms compared to treatment as usual (TAU) (Schuman et al., 2015; Slone et al., 2015). However, in another study, patients showed no improvement in symptoms, but followed fewer treatments, which could mean that FIGT leads to more efficiency of group therapy (Koementas-de Vos et al., 2018).

In all FIGT studies, patients completed standardized questionnaires before the start of each treatment session. But besides this similarity, there are mainly differences between the FIGT studies that could explain the mixed results. Variation exists in patient populations, treatment modalities, feedback instruments, instructions and training of therapists, whether or not discussing feedback results in the group, and in the (im)possibility of adjusting the group treatment when receiving feedback (Burlingame et al., 2018b; Davidsen et al., 2017; Hutson et al., 2020; Koementas-de Vos et al., 2018, 2022b; Newnham et al., 2010a; Schuman et al., 2015; Slone et al., 2015). It appears that an optimal way to use feedback in a group treatment setting has not yet been found and that most FIGT studies suggest the need for clear guidelines. More than a decade ago, the American Group Psychotherapy Association (AGPA) recommended the use of standardized group therapy questionnaires, the CORE-R battery, for the prevention of adverse outcomes in group psychotherapy settings (Strauss et al, 2008). The following primary tools were suggested: (1) the Group Therapy Questionnaire (GTQ) for group selection, (2) the Working alliance inventory (WAI) for process measurement, and (3) the Outcome Questionnaire 45 (OQ-45) for outcome measurement.

### Theory

Although it is still unclear what optimal use of feedback in group treatment would look like, in individual treatment settings it appears important to take contextual factors into account when using feedback. Sapyta et al. (2005) describe in their Contextual Feedback Intervention Theory (CFIT) that it matters what kind of feedback is given in which context. In addition, therapist factors, such as attitude towards using feedback, as well as patient factors (e.g., the severity of symptoms) can positively or negatively influence the efficacy of feedback (De Jong et al., 2018; Lewis et al., 2019).

In the context of group psychotherapy, it is reasonable to infer that therapist and patient factors as well as group-specific therapeutic elements are associated with the effectiveness of feedback. Based on a qualitative study on experiences and needs of FIGT by patients and therapists (Koementas-de Vos, et al., 2022a), it seems that these factors indeed play a role. Both patients and therapists experience greater insight into treatment progress and improved working alliance when they both receive and discuss feedback in the treatment sessions. Moreover, both patients and therapists prefer personalized treatment goals in addition to a general outcome measure, so that feedback is more personal and tailored to the treatment phase. Regarding group therapeutic factors, therapists and patients seem to have different experiences when using feedback in the group. Patients are positive about discussing feedback in the group, allowing them to learn from other group members, experience more cohesion and to feel more engaged. These aspects correspond to specific group therapeutic factors, as described by Yalom and Leszcz (2020): interpersonal learning, group cohesion and engagement. In contrast, therapists are concerned about possible negative effects on outcomes when discussing feedback in the group, like potentially negative competitive feelings in group members because of social comparison. Moreover, group therapists tend to discuss the feedback results to a lesser extent with NOT patients than with OT patients, while patients who are more involved in discussing the feedback in the group make more progress (Hutson et al., 2020). This suggests that patients who need feedback the most benefit from it the least in group treatment. In line with the theory of CFIT (Sapyta et al., 2005), it seems that patients’ and therapists’ attitudes towards feedback seem important in feedback-informed group treatment and that therapist, patients and group therapeutic factors should be taken into account, so that FIGT can be used optimally.

### Current Study: Renewed FIGT

Based on these earlier findings we developed a renewed FIGT tool: personalized goals were added to feedback with the OQ-45 (as recommended by the AGPA), therapists received training, coaching and intervision to support them in effectively using feedback in the group, and therapists were instructed to discuss the feedback actively in and with the group. The aim of this pilot study is to investigate the feasibility and acceptability of this new FIGT tool, as well as preliminary effects on clinical outcomes and processes. The results are compared to historical cohorts using an earlier version of a FIGT instrument (only OQ-45 feedback and no training or supervision for therapists) or no feedback at all.

It was hypothesized that the renewed FIGT tool is feasible and acceptable to both patients and therapists, and is more feasible and acceptable than an earlier version of FIGT. We used predetermined cut-off scores for detecting sufficient feasibility (dropout, attendance, feedback response and feedback discussions) and acceptability (usability by patients, usability by therapists, attitudes towards feedback by therapists). For example, feasibility was regarded as sufficient if percentages of dropouts and attendance rate were within the range of earlier studies in group treatment: dropout rates between 15 and 25% (Barkowski et al., 2020; Fernandez et al., 2015; Hans & Hiller, 2013) and attendance rates between 44 and 96% (Koementas-de Vos et al., 2022b; Schuman et al., 2015). We then compared the results on feasibility and acceptability to earlier cohorts. We also hypothesized that the renewed FIGT tool is more effective on symptoms and quality of life change, as well as on improving group therapeutic factors, than a previous FIGT tool and no feedback at all.

## Method

### Overview

This study is part of a larger research project on FIGT at GGZ-Noord-Holland Noord, a medium-sized mental health care institution in the Netherlands. Approval was granted by the internal research committee of the mental health institution. The organization’s privacy-protocol, based on the General Data Protection Regulation 2016/679 (The European Parliament and the Council, 2016) is followed.

### Design

The study has a quasi-experimental design in which Interpersonal Group Psychotherapy Groups (IPT-G) and Cognitive Behavioural Group Therapy Groups (CBT-G) for patients with anxiety and/or depressive mood disorders were included from September 2022 to April 2023. All these groups used a web-based renewed FIGT tool.

The collected data are compared with data of the following previous cohorts:

1. A TAU cohort between 2013 and 2015 in which data were collected on working alliance, group cohesion and group climate in patients (*N* = 70) who followed IPT-G or CBT-G without the use of feedback (Koementas-de Vos et al., 2018);
2. A TAU cohort between 2018 and 2021 in which data were collected on outcomes of patients (*N* = 93) who followed IPT-G or CBT-G without feedback (Koementas-de Vos et al., 2022b);
3. An OQ-45 cohort between 2018 and 2021 in which data were collected on feasibility, acceptability and outcomes of only OQ-45 feedback in patients (*N* = 104) who followed IPT-G or CBT-G with OQ-45 feedback (Koementas-de Vos et al., 2022b);
4. A therapist group between 2018 and 2021 in which data were collected on acceptability by therapists (*N* = 25) who have provided IPT-G or CBT-G with OQ-45 feedback (Koementas-de Vos et al., in 2022b).

### Participants

*Patients* In this manuscript, we describe only the inclusion of patients who followed IPT-G or CBT-G with the renewed FIGT tool between 2022 and 2023. For a detailed description of inclusion of patients in the other cohorts, see the publications of the earlier studies (Koementas-de Vos et al., 2018, 2022b). Patients in this study were eligible if they followed IPT-G or CBT-G between September 2022 and April 2023 with the renewed version of feedback. IPT-G was a half-open semi-structured psychotherapy group for patients with major depressive disorders. Patients could enter and exit IPT-G every eight sessions and follow up to 24 sessions. CBT-G was a closed, semi-structured psychotherapy group for patients with major depressive and anxiety disorders, with a maximum of 14 sessions. The duration of all group therapies varied between 8 and 24 sessions, with a frequency of one session per week. The minimum number of participants per group was four and the maximum was eight.

Inclusion and exclusion criteria for patients were concurrent with the criteria for participation in IPT-G or CBT-G. Inclusion criteria were: a main diagnosis of major depressive disorder or an anxiety disorder based on the criteria of the Diagnostic and Statistical Manual of Mental Disorders, Fifth Edition (DSM-5; American Psychiatric Association, 2013), the ability to formulate a treatment goal, age between 18 and 65 years, an IQ above 80 and absence of substance abuse or dependence. Exclusion criteria were: lack of motivation, acute psychotic or (hypo)manic symptoms and a severe suicide risk.

*Therapists* As with the patients, we only describe the participating therapists who used the renewed FIGT tool between 2022 and 2023. A detailed description of the therapists who used the only OQ-45 feedback between 2015 and 2018 can be found in the publication of Koementas-de Vos and colleagues (2022b). All therapists of the IPT-G and CBT-G groups between 2022 and 2023 were asked to participate and all agreed to do so. All groups in the current study were led by an experienced clinical or mental health psychologist or cognitive behavioral therapist accompanied by a psychotherapist, mental health psychologist in training for clinical psychologist, mental health psychologist, a psychologist with a master’s degree in training for mental health psychologist or psychologist with a master’s degree. In total, 15 therapists participated in the study, two male and 13 female, with a mean age of 41.5 years (SD = 13.1 years) and mean experience as a group therapist was on average 9.3 years (SD = 7.9 years). One mental health psychologist dropped out during the study because she decided to take on other tasks in the organization. She was replaced after one group treatment by another mental health psychologist who had not followed the training, but did receive coaching and intervision sessions.

### Feedback

Renewed feedback consisted of three adjustments compared to the previous web-based FIGT tool as described by Koementas-de Vos and colleagues (2022a): (1) monitoring progress on personalized treatment goals along with general functioning, (2) training, coaching and intervision for therapists, and (3) instructions for therapists to actively use the feedback with the group using a projector.

**Fig. 1:**
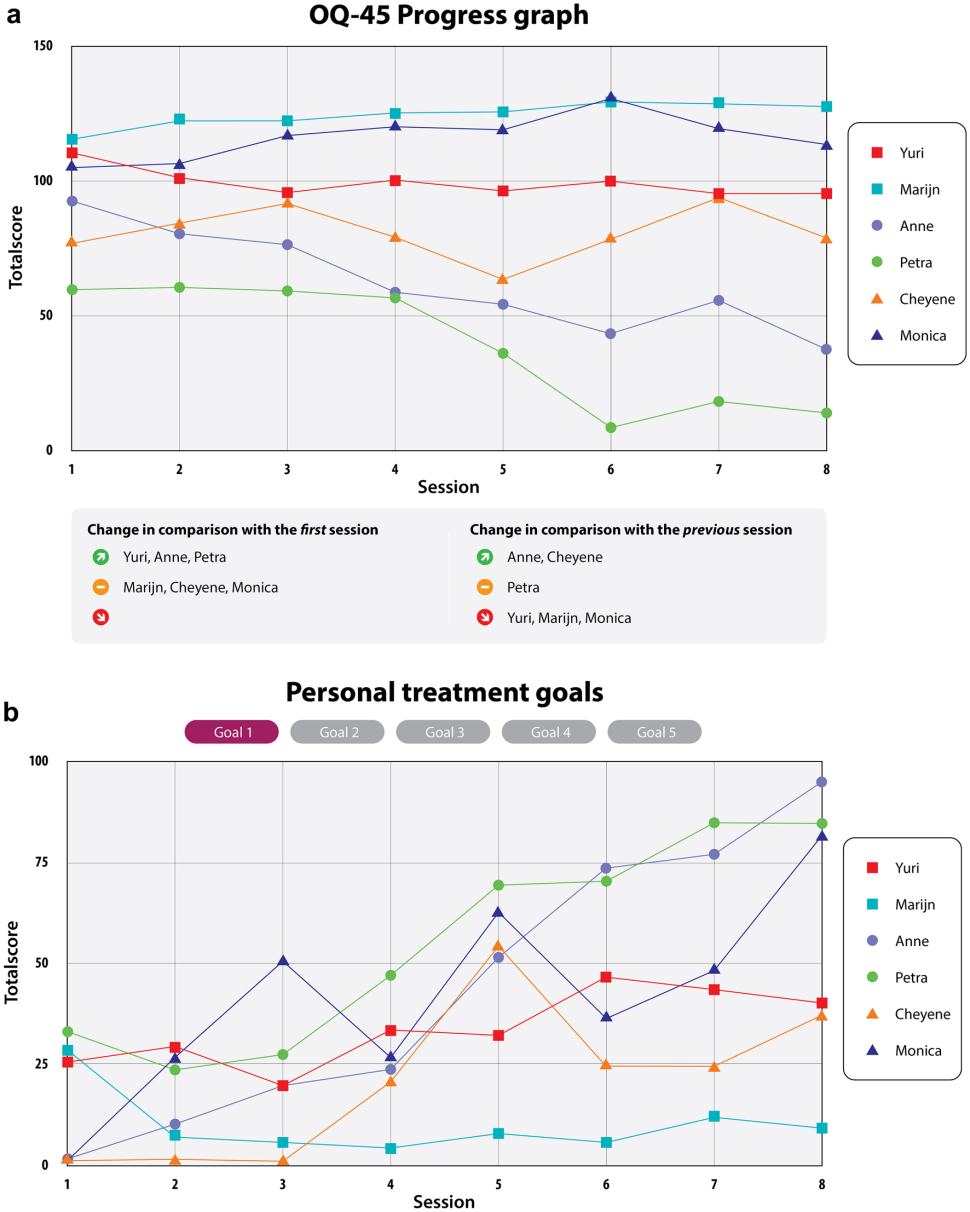
An example of feedback group graphs of the OQ-45 and personal treatment goals for therapists

*Web-based FIGT tool* For monitoring treatment trajectories, a web-based application has been developed, called Re:sponse©. It provides a digital dashboard for patients and therapists in group treatment. For this study, the Dutch version of the Outcome Questionnaire-45 (OQ-45, Lambert, 2017; De Jong et al, 2008) and personalized treatment goals were used as feedback instruments. The OQ-45 is a self-report questionnaire that measures a patient’s past week’s functioning based on 45 items. One day before each group session, patients completed the OQ-45. Both patients and therapists were able to see the results directly on a dashboard, with the difference that patients could only see their own trajectory and therapists could see the results of the group as a whole with the ability to monitor each patient’s results in more detail. For a detailed description of the feedback tool with the OQ-45, see the manuscript of Koementas-de Vos et al. (2022b). Along with the OQ-45, patients could independently set up to five goals and fill in their weekly progression on a VAS scale from 0 (not achieved at all) to 100 (completely achieved). Patients had the option to indicate the order of priority, with treatment goal 1 being the most important goal and treatment goal 5 as the least important. As with the OQ-45, progress was displayed in a graph on the dashboard. There was also a group chart for therapists showing the progress of all patients on all goals. See Fig. 1 for an example of therapist group graphs for both the OQ-45 and personal treatment goals.

*Training therapists* Prior to the first group session, each therapist completed a three hour group training on how to use feedback which was led by a feedback coach and group therapy supervisor. The following topics were discussed: (1) basic concepts of group dynamics, group therapy and group interventions; (2) own experiences with progress feedback and goal setting so far; (3) theory and research results about progress feedback, goal setting and group psychotherapy; (4) instructions on using the webbased FIGT tool Re:sponse©; (5) examples of how progress feedback can be used with the instruction to use the feedback actively in group therapy by checking the results prior to each session, discussing the results in the group by using a projector or big screen and promoting group engagement; (6) practice with the coaching method, and; (7) setting goals for following coaching sessions.

*Coaching* After the training, each group therapist couple had a monthly coaching session by a trained coach. In the months of September, October, November, January and February each group therapist couple received a 45-min coaching session. We chose for coaching instead of supervision, because we assumed that therapists had sufficient skills to lead group therapy, but using a new intervention such as the FIGT tool seems to lead therapists to think that their skills are underdeveloped and/or skills are not coming forward when they are needed. Coaching is a goal-oriented form of supervision, in which the coach has a facilitating style characterized by listening and asking questions instead of adopting a directive style as an expert (Cannon et al., 2021). Coaching aims to help a coachee to increase and develop his or her current skills, thereby initiating intentional behavioral change (Grant, 2013). Coaching can thus help the group therapists to learn to use their own problem-solving skills, to become more aware of existing skills and ultimately to gain more confidence in using feedback in group therapy. Prior to each coaching session, therapists send a sample of feedback from the web-based FIGT tool to the coach, accompanied by a completed coaching form, see Appendix 1 for an example. The coach used this information as a guideline for the conversation and followed a format for the coaching session based on the G.R.O.W. model developed by Whitmore (1992). G.R.O.W. stands for goals, reality, options or obstacles and way forward. In this study, we developed an instruction for coaches to structure each coaching session by the G.R.O.W. model with specific questions about group dynamics. See Appendix 2 for the developed G.R.O.W. format for FIGT coaching.

*Intervision* In December and March there was a 90-min intervision session with all group therapists together, also led by the feedback coach and group therapy supervisor. In both sessions, experiences with using feedback in group treatment were shared between group therapists using an intervision format. Furthermore, in the first intervision session the G.R.O.W. model was explained so that group therapists could practice using this model when discussing feedback with their co-therapist. In the coaching sessions after the first intervision, the therapists were supported by the coaches to question their co-therapists with the use of the G.R.O.W. model.

### Feasibility and Acceptability Measures

*Feasibility: dropout and attendance* To test the feasibility of the FIGT-tool, the number of dropouts and attendance rates were collected. A dropout was defined as a patient discontinuing therapy prematurely. In the half-open semi-structured IPT-G, a patient who stopped treatment before the last (eighth) session of a block counted as a dropout. The attendance rate was calculated by the number of actual sessions divided by the number of sessions offered × 100. Feasibility was sufficient if percentages of dropouts and attendance rate were within the range of earlier studies in group treatment: dropout rates between 15 and 25% (Barkowski et al., 2020; Fernandez et al., 2015; Hans & Hiller, 2013) and attendance rates between 44 and 96% (Koementas-de Vos et al., 2022b; Schuman et al., 2015).

*Feasibility: feedback response rate, and number of feedback discussions* To test for other aspects of feasibility, the feedback response rate and the number of feedback discussions were calculated. First, the feedback response rate, or proportion of feedback discussions conducted, was calculated by the number of completed questionnaires divided by the number of questionnaires offered × 100. Furthermore, each week patients were asked: “Did your therapist discuss the results of the progress feedback with you?” with the answer options yes or no. The number of confirmed responses to the additional question was divided by the number of completed questionnaires × 100. We selected 70% as an estimated threshold based on two FIGT studies that reported rates between 70 and 90% in which patients completed weekly questionnaires (Hutson et al., 2020; Koementas-de Vos et al., 2018), other FIGT studies did not report completion rates.

*Acceptability: usability experienced by patients* A similar 13-item user experience questionnaire for patients was used as in the OQ-45 feedback study between 2018 and 2021 (Koementas-de Vos et al., 2022b). This questionnaire measured how patients rated the FIGT tool on usability, relevance, reliability, specificity, completeness, effectiveness and future use. An example item is “Did the feedback give you insight in your treatment progress?”. The first 12 items were measured on a five-point rating scale ranging from never/not at all (0) to a lot/often (4), except for the items 1 and 2 that could be rated with yes or no. When more than 70% of the patients scored higher than a two (neutral) on an item, it was concluded that the acceptability of the tool was sufficient on that aspect. Item 13 was a multiple choice question with five answer possibilities:”What would you like to change about the feedback tool?” (a) nothing, (b) additional questionnaires to explore the causes of my symptoms, (c) symptom specific questionnaires, (d) an open field to write to my therapist, and (e) more tips on how to achieve my goals in the group.

*Acceptability: usability experienced by therapists* A similar nine-item user questionnaire for therapists was used as in the OQ-45 feedback study between 2018 and 2021 (Koementas-de Vos et al., 2022b). This questionnaire assessed how therapists rated the FIGT tool on helpfulness, relevance, clarity, specificity, usefulness and helpfulness to patients and future use. An example item is: “6. How specific did you find the feedback given?”. A similar five-point scale was used, ranging from never/not at all (0) to a lot/often (4), also except for the items 1 and 2 with the answer options yes or no. When more than 70% of the therapists scored higher than a two (neutral) on an item, it was also concluded that the acceptability of the tool was sufficient on that aspect.

*Acceptability: attitudes towards using FIGT* Attitudes towards feedback were measured by a part of an adapted 30-item questionnaire developed by De Jong et al. (2012), based on the CFIT (Contextual Feedback Intervention Theory) User Survey, designed by the Centre for Evaluation and Program Improvement of Vanderbilt University. To measure attitudes towards using feedback in group therapy, 13 items with a five-point scale ranging from strongly disagree (0) to strongly agree (4) were constructed. A sample item is: “I believe the feedback based on progress on personal goals and the OQ-45 is helpful for my group therapy.” The minimum total score was 0 and the maximum total score was 52, a high score meaning positive attitudes towards using FIGT.

### Clinical Outcome Measures

To assess the effectiveness of the FIGT-tool, the Depression Anxiety Stress Scale 21 Revised (DASS-21-R; De Beurs et al., 2001), and the MANchester Short Assessment of quality of life (MANSA, Priebe et al., 1999; Dutch translation; van Nieuwenhuizen et al., 2017) were selected.

The DASS-21-R is a 21-item self-report scale that assesses levels of depression, stress, and anxiety. It contains three seven-item scales (Depression, Stress and Anxiety) and each item has four response options ranging from 0 (did not apply to me at all) to 3 (applied to me much, or most of the time). Scales are scored independently and each scale has a maximum score of 42 (i.e., each scale is multiplied by 2 to make scores comparable to the DASS-42). There is no total score and a higher score on a scale indicates higher levels of depression, anxiety or stress. An example item of the depression scale is “I felt that I had nothing to look forward to”. The DASS-21-R has been validated for the Dutch population and has good psychometric properties. In the current study, Cronbach’s alphas for the subscales were good: 0.91 (Depression), 0.83 (Anxiety) and 0.84 (Stress).

The MANSA is a self-report questionnaire that measures the experienced quality of life by patients. The questionnaire has three sections and for this this study we only used the 16 items of the last section. Of these 16 items, four can be answered with yes or no. They assess the existence of close friends, number of contacts with friends per week, accusation of a crime and victimization of physical violence. The other items measure satisfaction with life as a whole, job (or sheltered employment such as performing work that is adapted to someone’s disabilities, or training/education, or unemployment/retirement), financial situation, number and quality of friendships, leisure activities, accommodation, personal safety, people that the patient lives with (or living alone), sex life, relationship with family, physical health, and mental health. Similar to the earlier studies, we only used the scores of the twelve satisfaction of life items to obtain a total score of quality of life. Each item contained a 7-point rating scale (1 = extremely negative, 7 = extremely positive) and the range of the total score was 12–84. An example item is “How satisfied are you with your physical health?” The internal consistency of the MANSA is found to be sufficient (α = 0.74; Priebe et al., 1999) to good (α = 0.81; Björkman & Svensson, 2005). The Cronbach’s alpha coefficient in this study was 0.78.

### Process Measures

The Dutch translation of the Working Alliance Inventory Short Form (WAI-S, Stinckens et al., 2009; Tracey & Kokotovic, 1989), Group Cohesion Questionnaire 23 (GCQ-23, Trijsburg, 2006) and Group Climate Questionnaire (GCQ-S, MacKenzie, 1983) were selected to measure therapy processes in the group treatment.

The WAI-S was used to assess the working alliance as experienced by patients with their clinicians. The WAI-S is a 12-item questionnaire with a 5-point Likert-scale (1 = never, 6 = always). There are three scales: Task (four items), Goal (four items) and Bond (four items). There is no overall score. Samples of items per subscale are “My therapist and I agree on what is important for me to work on” (Task), “As a result of these sessions I am clearer as to how I might be able to change” (Goal), and “My therapist and I respect each other” (Bond). The psychometric properties of the Dutch version of the WAI-S are good (Stinckens et al., 2009). The Cronbach’s alphas in this study were also good: 0.86 (Task), 0.91 (Goal) and 0.83 (Bond).

The GCQ-23 is a 23-item questionnaire based on the Group Attitude Scale and the Three-Factor Group Questionnaire described by Trijsburg (2006). The GCQ-23 has a six-point Likert-scale (0 = strongly disagree, 5 = strongly disagree) and consists of four scales: Bond with the group as a whole (Bond-G, seven items), Bond between members (Bond-M, four items), Cooperativeness (CO, four items) and Instrumental Value (IV, seven items). The total score is based on scores of 22 items. Example of items are “I like the group” (Bond-G), “There are group members that I like a lot” (Bond-M), “We cooperate and work together in the group” (CO) and “The group helps me to achieve my goals” (IV). The internal consistency and the test–retest reliability are good (Trijsburg et al., 2004). Cronbach’s alpha for the total score in the current study was good and for the subscales moderate to good: 0.94 (Total score), 0.81 (Band-G), 0.82 (Band-M), 0.60 (CO) and 0.95 (IV).

The GCQ-S consists of twelve items on a 7-point Likert-scale (0 = strongly disagree, 6 = strongly agree). There are three scales: Engagement (five items), Avoiding (four items) and Conflict (three items). There is no overall score. Examples of items on the subscales are “The members liked and cared about each other” (Engagement), “The members avoided looking at important issues going on between themselves” (Avoidance), and “There was friction and anger between the members” (Conflict). Internal consistencies of the subscales in an earlier study were good (Kivlighan & Goldfine, 1991), but in our study they were questionable to poor: 0.56 (Engagement), 0.48 (Avoidance) and 0.26 (Conflict). Koementas-de Vos et al. (2018) also found low internal consistencies in their FIGT study: 67 (Engagement), 0.54 (Avoidance), and 0.50 (Conflict).

### Procedure

Before the three hour training, therapists filled out the attitudes towards FIGT questionnaire. Therapists then performed intakes for the IPT-G or CBT-G and asked their patients to participate in the study. Patients received an informed consent form and had at least one week’s time to decide whether they wanted to participate or not. When patients did not wish to participate, they did not fill out pre- and post-measurements, but they were still able to use the feedback tool and participate in the group treatment. Before the start of the first session, participating patients received an e-mail with a link to complete the DASS-21-R and MANSA. Prior to each session, all patients received an email to fill out their progress on the feedback instruments each session. Prior to the last session, participating patients were asked to fill out the DASS-21-R and the MANSA again along with the user experience questionnaire, WAI-S, GCQ-23 and the GCQ-S. At the end of the study, therapists were asked to fill out the number of dropout and attendance, as well as the user experience and attitudes towards FIGT questionnaires. See Table 1 for a visualization of the administration of the instruments.

**Table 1:** Administration of instruments by therapists and patients at different moments during the study

Measure	Before group treatment	After group treatment
*Therapists*		
Dropout and attendance rates		X
User experience questionnaire		X
Attitudes towards FIGT	X	X
*Patients*		X
User experience questionnaire DASS-21-R + MANSA		X
WAI-S, GCQ-23 + GCQ-S	X	X

### Data-Analysis

IBM SPSS (version 29) was used for all data-analyses, a p value < 0.05 was considered significant for all tests. Pre-treatment differences between the improved feedback cohort and earlier cohorts were tested on participant characteristics: the chi-square test was conducted for categorical variables and the t-test for continuous variables. When the assumptions underlying these tests were not met, we opted to utilize the nonparametric Mann–Whitney U test as an alternative statistical approach.

*Feasibility: dropout and attendance* Feasibility was sufficient if percentages of dropouts were between 15 and 25% and attendance rates between 44 and 96%, using descriptive analyses. For comparison with previous cohorts, the Kruskall Wallis test was performed, because the assumption of normal distribution was violated.

*Feasibility: feedback response rate and number of feedback discussions* Feedback response rate and confirmed feedback discussions were tested against the aimed threshold of 70% performing descriptive analysis. For the comparison with the earlier OQ-45 cohort in 2018–2021, we performed the non-parametric Mann–Whitney U test.

*Acceptability* Concerning the acceptance of the FIGT tool, a descriptive analysis was applied. Furthermore, the scores on the items were compared to the scores of patients and therapists in the OQ-45 cohort of 2018–2021. A non-parametric Mann–Whitney U test was conducted.

*Acceptability: attitudes towards using FIGT* The total scores on attitudes towards using FIGT questionnaire rated by therapists at the end of the study were compared to total scores at the start of the study using the nonparametric Wilcoxon test.

*Clinical outcomes* We performed a two-level multilevel analysis (MLA; Level 1 = patient; level 2 = group) to test if the DASS-21-R and MANSA scores of patients in the current study showed more improvement than in the OQ-45 feedback cohort in 2018–2021 and the TAU cohort without feedback in 2018–2021. We postulated an unconditional model with the total scores of the DASS-21-R and MANSA as dependent variables. Then we added research condition to test if the scores were significantly different between the renewed feedback condition in comparison to both the OQ-45 only condition and TAU condition. In the analysis, we controlled for pretreatment differences by adding variables on which significant differences had been found, as covariates in the multilevel model. These variables were education, ethnicity, marital status and treatment modality. If a parameter improved the fit of the model, it was maintained. If not, the variable was removed. After each change in the model, the difference was tested with the chi-square statistic. In the analysis of the stress and anxiety subscales of the DASS-21-R, as well as the total score of the MANSA, the inclusion of the Time in Days variable, after accounting for covariates, demonstrated an improved model fit. However, in the case of the depression subscale of the DASS-21-R, a better model fit was achieved by incorporating both the Time in Days and subsequent Condition variables.

*Process measures: WAI-S, GCQ-23 and GCQ-S* An ANCOVA was conducted to compare the scores on the WAI-S, GCQ-23 and GCQ-S between the current cohort and the control cohort in 2013–2015. We added the covariates primary diagnosis, education and treatment modality (IPT/CBT) to control for pretreatment differences. We did not choose an MLA because there was only one measuring moment, namely at the end of the treatment, meaning there was no benefit to using MLA.

*Effect sizes:* We added effect sizes in the Results section based on the article of Tomczak and Tomczak (2014). We only reported the confidence intervals for the parametric tests with normal distributions, as described by Lee (2016). For the MLA, effect sizes were calculated using the following formula:

$$d = \frac{{{\text{estimate}}_{t} - {\text{estimate}}_{{pretreatment}} }}{{{\text{sd}}_{{pretreatment}} }}$$

## Results

### Preliminary Analysis

Between September 2022 and April 2023, two half-open IPT groups and five closed CBT groups were organized with 72 patients receiving group treatment with renewed FIGT. Of the 72 patients, five patients were excluded because they did not meet the inclusion criteria, for example they were classified with another main diagnosis than a major depressive disorder or anxiety disorder. Two patients declined to participate in the study, leaving a total of 65 patients (90%).

In Table 2 the characteristics of the current cohort with renewed FIGT, the TAU cohort between 2013 and 2015, the TAU cohort 2018–2021 and the OQ-45 cohort between 2018 and 2021 are described. There were no significant differences between the current cohort and other cohorts with regard to gender, age, employment and comorbid DSM-5 classification. Also, there were no differences between the current cohort and the TAU or OQ-45 cohort between 2018 and 2021 on DASS-21-R and MANSA pretreatment scores. The TAU cohort of 2013–2015 had no DASS-21-R and MANSA pretreatment score, because in that study the OQ-45 was, apart from the feedback measure, also the outcome variable. As can be seen in Table 2, compared to the TAU cohort in 2013–2015, in the current cohort there were significantly fewer patients with an anxiety disorder, fewer patients with a high education level and fewer patients with IPT-G. Furthermore, in comparison to the TAU cohort in 2018–2015, in the current cohort there were significantly fewer patients with a high education level and fewer patients who followed IPT-G. Compared to the OQ-45 cohort in 2018–2021, in the current cohort there were relatively more patients with a indigenous origin and a single status.

**Table 2:** Patient characteristics of current renewed FIGT cohort in comparison with earlier cohorts

	TAU 2013–2015	TAU 2018–2021	OQ-45 2018–2021	New FIGT 2023
	(*n* = 70)	(*n* = 93)	(*n* = 104)	(*n* = 65)
	*n* (%)/Mean ± SD	(%)/Mean ± SD	(%)/Mean ± SD	*n* (%)/Mean ± SD
Gender				
Male	30 (43%)	26 (28%)	46 (44%)	27 (42%)
Female	40 (57%)	67 (72%)	58 (56%)	38 (58%)
Age (years)	40.0 ± 13.0	40.6 ± 13.3	41.2 ± 13.5	36.9 ± 13.4
Primary diagnosis				
Major depressive disorder	42 (60%)*	75 (81%)	85 (82%)	51 (78%)
Anxiety disorder	28 (40%)	18 (19%)	19 (18%)	14 (22%)
Ethnicity				
Indigenous origin	69 (99%)	87(94%)	92 (89%)*	64 (98%)
Employed	37 (53%)	46 (50%)	54 (52%)	27 (42%)
Education				
Low	12 (17%)*	5 (5%)*	13 (13%)	4 (6%)
Intermediate	26 (37%)	46 49%)	58 (56%)	41 (63%)
High	32 (46%)	42 (45%)	33 (31%)	18 (28%)
Marital status				
Single	26 (37%)	36 (39%)	37 (36%)*	32 (49%)
Married	16 23%)	27 (29%)	24 (23%)	14 (22%)
Other	28 (40%)	30 (32%)	43 (41%)	19 (9%)
Comorbid DSM-5 disorder	34 (49%)	48 (52%)	49 (47%)	28 (43%)
Type of Psychotherapy				
IPT	53 (76%)*	25 (27%)*	47 (45%)	28 (43%)
CBT	17 (24%)	68 (73%)	57 (55%)	37 (57%)
DASS-21-R pretest score				
Depression	n/a	10.95 ± 4.96	11.06 ± 5.36	10.72 ± 4.60
Anxiety	n/a	7.77 ± 4.23	7.92 ± 4.42	7.62 ± 4.64
Stress	n/a	10.38 ± 4.32	11.06 ± 4.61	10.40 ± 4.44
MANSA pretest score				
Total score	n/a	47.46 ± 7.29	47.17 ± 9.10	46.93 ± 8.77

*p < 0.05 in comparison to new FIGT cohort

**p < 0.01 in comparison to new FIGT cohort

### Feasibility

#### Feasibility: Dropout and Attendance

Of the 65 patients, one dropped out before the start of the first session and 12 patients dropped out during the group, meaning 18.5% of the patients dropped out during therapy, which is within the range of 15–25%. There were several reasons for dropping out, such as missing too many sessions because of other appointments, not feeling comfortable in a group setting, avoidance of confronting themes and somatic illness. Therapists were instructed to ask all patients if dropping out was related to the use of feedback, but this was never the case. Compared to the dropout rates of the TAU (13%) and OQ-45 cohort (8%) between 2018 and 2021, dropout rates were not significantly higher in the current cohort, although a trend is visible contrasting with the hypothesis (*H* (2) = 5.367, *p* = 0.07, 95%, E^2^_*R*_ = 0.02). The attendance rate was 85% in the current cohort and was not significantly different from the attendance rates of the TAU and OQ-45 cohorts in 2018–2021. The attendance rate fell within the range of 44–96%, based on earlier FIGT (Koementas-de Vos et al., 2022b; Schuman et al., 2015). In the current cohort, the minimum number of sessions attended was two and the maximum was 18 with a mean of 9.7 sessions (SD = 3.8).

#### Feasibility: Feedback Response Rate and Percentage Confirmed Feedback Discussions

In the current cohort, 66% of the feedback measures were completed by patients. This is below the 70% cut off. Of the 52 patients, 43% completed at least 70% of the OQ-45s offered, 19% completed all OQ-45s and 6% of the patients did not fill out any of the OQ-45s offered. Patients completed on average 9.9 questionnaires (SD = 3.74) during group treatment. The feedback response rate of 66% did not differ significantly from the response rate of the OQ-45 cohort in 2018–2021, which was 68%.

Patients reported that on average 73% of the OQ-45s and progress on personal treatment goals were discussed with them, which is above the cutoff of 70%. Furthermore, 10% of the patients said that the OQ-45 results were discussed with them every session, and 8% of the patients reported that the OQ-45 results were never discussed with them. The percentage of confirmed feedback discussions was significantly higher than in the OQ-45 cohort in 2018–2021 (*U* (N_OQ-45_only_ = 82, N_OQ-45+_ = 48) = 2613.50, *z* = 3.12, *p* < 0.01, *r*^2^ = 0.07), with a median in the current cohort of 79% confirmed feedback discussions and in the OQ-45 cohort in 2018–2021 the median was 67%.

### Acceptability

#### Acceptability by Patients

42 of the 65 patients filled out the 13-item user experience questionnaire at the end of group treatment. As can be seen in Table 3, 79% of the patients reported that they filled out the OQ-45 every session (item 2), also 79% experienced that the feedback was discussed with them every session (item 3) and 71% of the patients would like to use the FIGT tool again in the future (item 12). It turned out that 91% of the patients who scored positive on item 2 also scored positive on item 3. Less than 70% of the patients scored positively on the other items, see Table 3. On item 13, when asked what needed to be improved, 26% answered that nothing should be changed, 26% wanted additional questionnaires to investigate the causes of their symptoms, 24% preferred a specific symptom questionnaire, 12% preferred an open text field to communicate with the therapist and 12% wanted tips to achieve treatment goals.

**Table 3:** Percentage of patients who scored 3 and 4 (meaning regularly/very much/yes) on the usability questionnaire in the current cohort and OQ-45 cohort in 2018–2021

	New FIGT 2023 *N* = 42	OQ-45 2018–2021 *N* = 61	*p*-value
1. Did you use the feedback tool?	57%	67%	.32
2. Did you fill out the OQ-45 every session?	79%	67%	< .01
3. Has the feedback been discussed with you every session?	79%	42%	< .01
4. Has the feedback given you insight in the treatment progress?	62%	37%	< .01
5. How relevant did you find the feedback given?	57%	37%	.04
6. How reliable did you find the feedback given?	57%	72%	< .01
7. How specific did you find the feedback?	50%	72%	< .01
8. To what extent was the feedback complete enough for you?	45%	56%	.65
9. To what extent contributed the feedback to effect of therapy?	48%	47%	.65
10. Has the feedback helped improve the relationship between you and your group therapists?	45%	47%	.87
11. Did the feedback lead to more agreement between you and your therapists about your treatment goals?	52%	41%	.049
12. Would you like to use the tool again in the future?	71%	30%	< .01

Compared to the 2018–2021 OQ-45 cohort, there were significant differences on items 2, 3, 4, 5, 6, 7, 11, and 12 with the patients in the current cohort, see Table 3. Consistent with the hypothesis, more patients with renewed FIGT reported having completed the OQ-45 each session, that the feedback was discussed them, that the feedback provided insight in the therapy process, that the feedback was perceived as relevant, that the feedback led to more agreement with their therapist on the therapy goals and the percentage of patients who wanted to use the FIGT tool in the future has doubled. Contrary to the hypothesis, fewer patients in the current cohort found the feedback to be reliable and specific.

#### Acceptability by Therapists

**Fig. 2:**
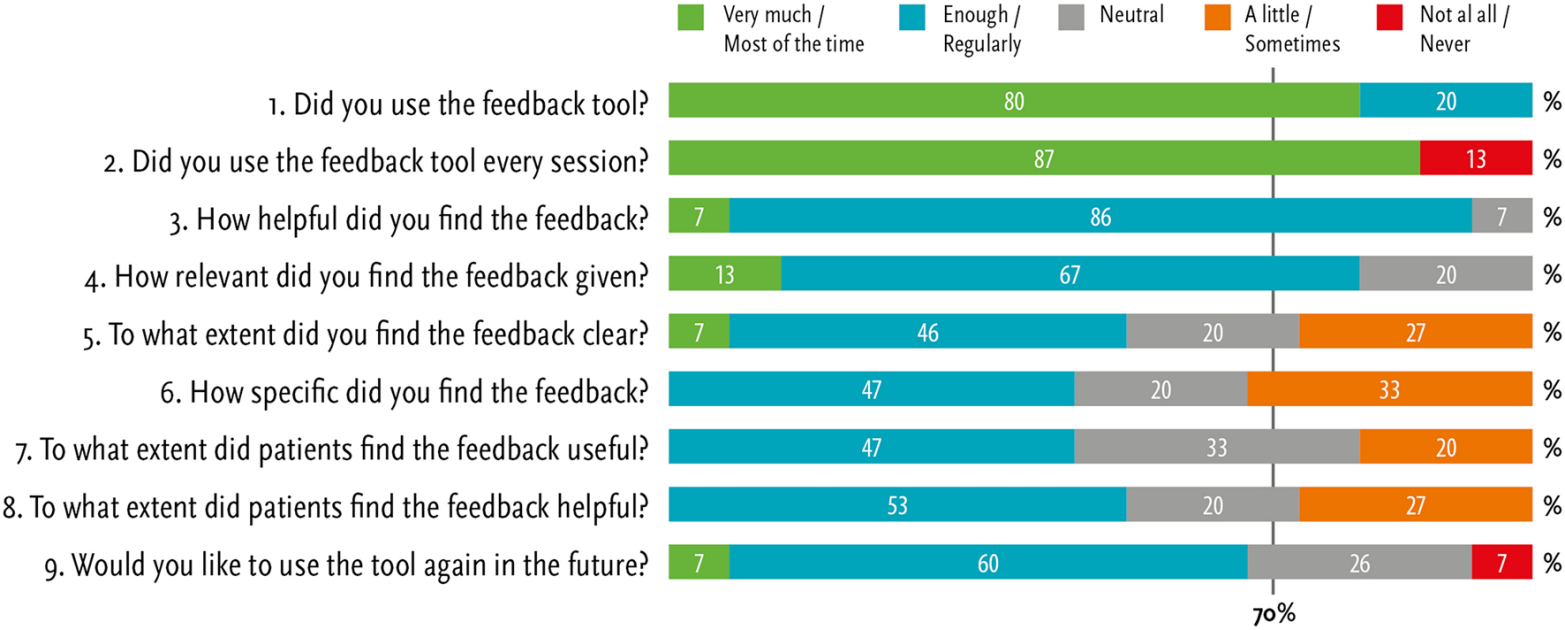
Acceptability of the renewed feedback tool rated by therapists at the end of the study (*N* = 15)

All therapists filled out the nine-item user questionnaire and all confirmed that they used the feedback regularly or always (item 1) and 87% used the FIGT tool every session. Furthermore, 93% reported the feedback tool as helpful (item 3) and 80% rated the feedback as relevant (item 4), as can be seen in Fig. 2. Less than 70% of the therapists scored positively on the other items. On item 10, when asked what needed improvement, four therapists did not respond, two therapists preferred symptom-specific questionnaires, and nine therapists suggested technical improvements to the feedback application, e.g., improvement of the VAS slider or that emails should not end up in the spam folder of patients.

In comparison to the responses of the therapists in the OQ-45 cohort 2018–2021, there were no significant differences on the nine-item user questionnaire with the responses of the therapists in the current cohort.

### Acceptability: Attitudes Towards Using FIGT

At the beginning of the study, 14 of the 15 therapists completed the questionnaire on attitudes toward the use of FIGT. The therapist who replaced the dropped-out therapist missed this assessment at the start of the study. The therapist who dropped out of the study was asked to complete the assessment at the end of the study, resulting in 15 of the 15 therapists completing the 30-item questionnaire at the end of the study.

The mean total score of attitude towards feedback in group therapy based on the CFIT questionnaire was 36.9 (SD = 5.4) at the start of the study and 34.5 (SD = 5.5) at the end of the study. A Wilcoxon signed rank test showed that there were no significant differences between the total score at the start (*Md* = 34.5, *N* = 14) and the total score at the end of the study (*Md* = 32.0, *N* = 15), although a trend was visible in contrast to the hypothesis (*p* = 0.07). When the individual items were compared between the start and the end of the study, it was found that at the end of the study therapists were less convinced that the scores on the personal treatment goals and OQ-45 were important factors to detect therapeutic change, with a large effect size (item 3; *Md*_*start*_ = 2*, Md*_*end*_ = 3, *z* = − 2.50, *p* = 0.01, *r* = − 0.75).

### Clinical Outcomes

#### Depression, Anxiety and Stress Symptoms

As can be seen in Table 4, separate multilevel analyses were performed for all the subscales of the DASS-21-R comparing three conditions: the current cohort, the TAU cohort in 2018–2021 and the OQ-45 cohort in 2018–2021. The variables ethnicity, education, marital status and treatment modality (IPT/CBT) were added as covariates in the multilevel model to control for pretreatment differences. In Table 5, the pretreatment and post treatment scores of the DASS-21-R subscales of all cohorts are presented. As can be seen, all patients of the three cohorts had improved at the end of treatment, but only the scores on subscale Depression were significantly improved. Furthermore, the interaction TAU Condition * Time for the subscale Depression significantly predicted depressive outcomes, but contrary to the hypothesis. This means that patients in the current renewed FIGT cohort showed less improvement in depressive symptoms compared to those in the TAU condition from 2018 to 2021 (95% CI of improvement of TAU in comparison to renewed FIGT: − 0.04 to − 0.00 per day). This difference was statistically significant, but with a small effect size, *t* = 2.081, *p* = 0.04. *d* = − 0.02.

**Table 4:** Fixed and Random Effect Estimates and standard errors for the Multilevel Linear Models of the DASS-21-R and MANSA comparing the TAU cohort with the OQ-45 + (current cohort) and OQ-45 (OQ-45 cohort 2018–2021)

Parameter	Estimates (standard error)	*p*-value
Depression		
Fixed effects		
Intercept (Depression total score)	14.006 (1.267)	< .01
Time in days	− .004 (.008)	< .01
TAU condition * Time in days	−.021 (.010)	.04
OQ-45 condition * Time in days	−.005 (.010)	.64
Random effects		
Residual	12.505 (1.324)	< .01
Patient intercept variance	11.920 (1.951)	< .01
Anxiety		
Fixed effects		
Intercept (Anxiety total score)	9.619 (1.123)	< .01
Time in days	−.011 (.006)	.08
TAU condition * Time in days	.079 (.734)	.92
OQ-45 condition * Time in days	−.006 (.714)	.99
Random effects		
Residual	7.479 (.815)	< .01
Patient intercept variance	10.909 (1.556)	< .01
Stress		
Fixed effects		
Intercept (Stress total score)	11.652 (1.059)	< .01
Time in days	−.007 (.007)	.36
TAU condition * Time in days	−.011 (.009)	.23
OQ-45 condition * Time in days	.005 (.009)	.53
Random effects		
Residual	9.730 (1.036)	< .01
Patient intercept variance	7.546 (1.380)	< .01
Quality of life		
Fixed effects		
Intercept (MANSA total score)	41.118 (2.328)	< 0.01
Time in days	.027 (.013)	< 0.01
TAU condition * Time in days	.020 (.016)	.19
OQ-45 condition * Time in days	−.007 (.015)	.66
Random effects		
Residual	25.374 (2.675)	< 0.01
Patient intercept variance	53.063 (6.418)	< 0.01

Covariates ethnicity, education, marital status and treatment modality are not reported in this table

**Table 5:** Posttest scores and difference scores (post treatment score minus pretreatment scores) on the DASS-21- R and MANSA with means and standard of the three conditions: New FIGT (current cohort), TAU (TAU cohort 2018–2021) and OQ-45 (OQ-45 cohort 2018–2021)

	New FIGT		TAU 2018–2021		OQ-45 2018–2021	
	Mean ± SD		Mean ± SD		Mean ± SD	
	Pretest	Posttest	Pretest	Posttest	Pretest	Posttest
DASS-21-R score						
Depression	10.72 ± 4.60	8.53 ± 5.64	10.95 ± 4.96	7.61 ± 5.30	11.06 ± 5.36	9.28 ± 5.75
Anxiety	7.62 ± 4.64	6.09 ± 4.45	7.77 ± 4.23	5.84 ± 3.91	7.92 ± 4.42	6.71 ± 4.55
Stress	10.40 ± 4.44	8.34 ± 4.69	10.38 ± 4.32	8.49 ± 3.87	11.06 ± 4.61	9.36 ± 4.47
MANSA score	46.93 ± 8.77	51.17 ± 10.15	47.46 ± 7.29	53.28 ± 9.55	47.17 ± 9.10	51.15 ± 10.12

#### Quality of Life

As can be seen in Table 4 and 5, all patients in the three cohorts had improved significantly on quality of life at the end of treatment. In contrast to the hypothesis, there were no significant differences on the MANSA between the current cohort and the TAU and OQ-45 cohorts in 2018–2021.

#### Process Factors

With regard to the WAV-12 outcomes, there were no differences on working alliance between the current cohort and the TAU cohort in 2013–2015 in terms of task, goals and bond. For the GCQ-23 scores, differences were found on the total score and the subscales Band-M and CO after correction for the pretreatment differences on the variables primary diagnosis, education and treatment modality (IPT/CBT), with medium effect sizes. This means that patients in the current cohort (M = 96.2, SD = 15.4, 95% CI 91.6–101.1) experienced more group cohesiveness in general at the end of group therapy than patients in the TAU cohort in 2013–2015 (M = 88.5, SD = 14.5, 95% CI 84.5–92.3), *F*(1,1508.965) = 7.254, *p* = 0.01, η^2^_*p*_ = 0.06). Also, patients in the current cohort (M = 17.0, SD = 3.9, 95% CI 15.7– 18.5) reported more bonding with the other group members than patients in the TAU cohort in 2013–2018 (M = 14.6, SD = 4.5, 95% CI 13.5–15.6), *F*(1,138.126) = 7.793, *p* = 0.01, η^2^_*p*_ = 0.08). Furthermore, patients in the current cohort (M = 17.2, SD = 2.8. 95% CI 16.2–18.1) rated the cooperativeness with the group higher than patients in the TAU cohort 2013–2018 (*M* = 15.3, SD = 2.96, 95% CI 14.6–16.0), *F*(1,96.666) = 11.647, *p* < 0.01, η^2^_*p*_ = *0.09*. On the GCQ-S, patients in the current cohort (M = 22.5, SD = 3.5, 95% CI 21.3– 23.6) reported a more positive working atmosphere on the subscale Engagement than patients in the TAU cohort 2013–2018 (M = 20.5, SD = 3.5, 95% CI 19.6–21.3), *F*(192.654) = 7.554, *p* < 0.01, η^2^_*p*_ = 0.07. In addition, patients in the current cohort (M = 3.2, SD = 2.3, 95% CI 2.1–4.1) experienced less anger and tension in the group, measured by the subscale Avoidance of the GCQ-S, than patients in the TAU cohort 2013–2018 (M = 5.6, SD = 3.5, 95% CI 4.9–6.4), *F*(1,137.891) = 14.423, *p* < 0.01, η^2^_*p*_ = 0.12.

## Discussion

This study investigated the feasibility, acceptability and outcomes on symptoms, quality of life and group therapeutic factors of a renewed version of FIGT for patients with anxiety and/or depressive disorders. Results showed that the renewed FIGT tool was more feasible, patients experienced more feedback discussions and rated it as more useful than patients who used a FIGT tool with the OQ-45 alone. 71% of the patients and 66% of the therapists would like to use the renewed FIGT tool in the future. Attitudes towards feedback were not improved at the end of the study and therapists were less convinced that the feedback instruments (personal treatment goals and OQ-45) adequately detected therapeutic change. Although feasibility and acceptability were improved, the renewed FIGT tool did not lead to more effectiveness on symptoms or quality of life. Compared to patients in group treatment without feedback, patients with renewed FIGT showed even less improvement on depressive symptoms but experienced more group cohesion, more engagement and less avoidance.

The results on outcomes are almost similar to an earlier FIGT study of Koementas-de Vos et al. (2022b) with only OQ-45 feedback, where also no improvement of effectiveness of FIGT was found and that feedback may even have adverse effects on improvement of depressive symptoms and quality of life. It was unexpected that the renewed FIGT tool did not show positive effects on symptoms and quality of life, because in this study extra attention was paid to the implementation barriers as described by Lewis et al. (2019): (1) the feedback was made more personal to the patient by adding personal treatment goals, patients filled out the questionnaires frequently and experienced more feedback discussions; (2) therapists received extra support with training, coaching and intervision and they actively used feedback in their treatment; and (3) the organization invested in training and coaching for the 15 therapists. Similar results were found by van Sonsbeek et al. (2023), who performed a study in individual treatment settings with extra attention on implementation strategies for using feedback, but did not find an effect on outcomes either. In comparison to the earlier study of Koementas-de Vos et al. (2022b), this study fortunately provides more information that could explain the unexpected results in FIGT, as patient, therapist, and group factors are examined.

With regard to patients’ factors, it is possible that the renewed FIGT tool still did not meet the needs of patients sufficiently. As described in the Contextual Feedback Theory by Sapyta et al. (2005), it is important that feedback should be accurate and fit one’s goals. As suggested in the earlier study of Koementas-de Vos et al. (2022b), patients with severe anxiety or depressive disorders with comorbid disorders may need other information than progress on general functioning alone. Although patient acceptability as well as the number of feedback discussions were improved with renewed FIGT compared to only OQ-45 feedback, it seems insufficient to improve the effectiveness. Furthermore, contrary to our expectations patients in our sample perceived the feedback as less reliable and specific compared to patients who had used the previous FIGT tool. They possibily needed additional feedback measures and/or more help from the therapists to benefit from FIGT. Almost 75% of the patients in this study would indeed prefer further improvements of the FIGT tool, such as additional questionnaires to investigate the causes of their symptoms, a specific symptom questionnaire, an open text field to communicate with their therapist and recommendations to achieve treatment goals. In addition to patient factors, therapist factors seem to play a role in the results as well. Therapists’ attitudes towards the use of feedback in group treatment was not improved at the end of the study and therapists even expressed more doubts regarding the instruments’ ability to effectively detect therapeutic changes. It is possible that therapists doubted the validity of the feedback which could have potentially led to its rejection or, at least, limited integration into their work for adjusting treatment interventions. Beliefs or attitudes about the emotional tone or validity of provided feedback are indeed crucial factors in utilizing feedback effectively (Herzog et al., 2023). It might be beneficial to provide therapists in IPT-G and CBT-G with additional support in detecting changes through the use of other appropriate questionnaires and in problem-solving when faced with stagnation or decline in their patients’ progress. Therapists indeed reported they struggled with stagnation of patients which made them feel insecure. Clinical support tools may help them to overcome these struggles, because they are designed to detect possible causes of stagnation or decline and give additional treatment recommendations. In individual settings, the effectiveness of feedback increases when these clinical support tools are used (De Jong et al., 2021). Another challenge for the therapists in this study was they felt they had little time to discuss the feedback results in the group. De Jong et al. (2023) propose a clinical troubleshooting method with six steps for investigating the causes of stagnation of patients who are not-on-track and to adjust the treatment plan. But in group therapy with an average of 6–8 patients, it is impossible to do this with several patients. A suggestion may be to add individual feedback sessions with patients who are not-on-track to explore plausible causes for stagnation or decline and adjust treatment if needed. Recently another study on FIGT for structured CBT groups proposed a similar suggestion (Gryesten et al., 2023). However, this contrasts with the preferences of patients who favor discussing feedback results within the group, as highlighted in the study by Koementas-de Vos et al. (2022a). Perhaps a compromise involving a combination of individual feedback sessions and less frequent group discussions on feedback could lead to more optimal effects.

Besides patient and therapist factors, it is clear that group therapeutic factors are related to the use of FIGT. In line with the qualitative study on the experiences and needs of patients and therapists in using FIGT (Koementas-de Vos et al., 2022a), it was observed that FIGT is associated with increased cohesiveness, greater engagement, and reduced avoidance. These outcomes were not found to be linked to improved clinical outcomes, which differs from the findings of most group treatment studies (Burlingame et al., 2018a). One explanation is that most therapy groups in the current study are short-term with a cognitive-behavioral orientation. It is found that these kind of task-oriented groups have lower correlations between cohesion and outcomes than long-term groups with an interpersonal focus or psychodynamic orientation (Burlingame et al., 2018a). Nonetheless, it appears that group therapeutic factors can be influenced by feedback, even in short-term, task-oriented groups like IPT-G and CBT-G. This may imply that individual change processes may require more time with FIGT because therapists and patients could be more focused on the group dynamic processes, and improvements in outcomes may manifest at a later stage. It is indeed found that the relationship between cohesion and outcomes is higher when groups are longer and is the highest in groups with > 20 sessions (Burlingame et al., 2018a). The duration of CBT-G in this study was no more than 14 sessions and the duration of IPT-G was 24 sessions, but due to the limited eight-month time frame of this study, there were no groups that lasted longer than 16 sessions. It is possible that if the study had run longer and CBT-G was extended to > 20 sessions, other results on outcomes could be found.

There are several limitations of this study that should be taken into account. In the first place, this is a quasi-experimental study that involves a comparison between patients in the current study cohort and those in previous studies (TAU and OQ-45 feedback). Consequently, randomization did not occur, leading to the possibility of systematic differences between subjects. The initial analysis indicated disparities in certain demographic variables between the cohorts, and we made efforts to reduce the effects of these differences as much as possible in our data analyses. Another major limitation is that large amounts of data are required in group therapy studies to achieve sufficient power to detect any effects, due to the interdependence between subjects within groups and the low effect sizes of feedback (around 0.15; de Jong et al., 2021). Additionally, we have incorporated multiple variables and conducted several analyses, which may increase the likelihood of type I errors. However, we did not correct for multiple testing due to the pilot nature of the study, aimed at deriving insights and knowledge from the outcomes. Another limitation is the low Cronbach’s alpha between 0.26 and 0.56 of the subscales of the Group Climate Questionnaire (GCQ-S). These low coefficients may be related to low internal consistency, but are also more commonly found when questionnaires or subscales are too short (Tavakol & Dennick, 2011). For example, the Conflict subscale consists of only three items which could possibly explain the low Cronbach’s alpha. Nevertheless, the results of the GCQ-S should be interpreted with caution. Furthermore, even with extra attention to implementation barriers, using feedback is generally not easy at first and it takes time to implement feedback in a group setting. The study lasted eight months and was possibly too short for therapists to become sufficiently familiar with the use of feedback. Therapists also indicated that it took time to find an acceptable way to apply feedback in a group setting.

In terms of implications, it appears that when implementation barriers at patient, therapist, and organizational levels as described by Lewis et al. (2019) are taken into account, it is possible to implement a FIGT tool that is more feasible and appreciated by most patients and therapists. For patients, it seems useful to monitor personal treatment goals in addition to general functioning. Moreover, group therapeutic factors appear to be positively related to the use of FIGT. However, based on this study, there seems to be a risk associated with the use of FIGT in more task-oriented short-term groups for patients with severe anxiety and depressive disorders: a more feasible and useful FIGT tool alone is not sufficient to improve the effectiveness of short-term group therapy. It seems too early to conclude that FIGT should no longer be used for this group of patients in IPT-G or CBT-G, because the positive effects on group cohesion and group climate can be predictors of therapeutic change, as described in the meta-analysis of Burlingame et al. (2018a). Therefore, our proposal is to further investigate the long-term effects of FIGT, to further adapt the FIGT tool to the needs of patients and therapists, to study the addition of a clinical support tool for therapists and experiment with different type of feedback sessions with patients (individually and/or in the group).

In conclusion, this study shows that renewed FIGT was more feasible and acceptable, and appeared to have positive effects on patients’ acceptability, cohesion and the group climate. The renewed FIGT tool did not improve the effectiveness on symptoms and quality of life in comparison to no feedback or only OQ-45 feedback and may have adverse effects on the effectiveness on depressive symptoms. In line with the Contextual Feedback Intervention Theory (CFIT; Sapyta et al., 2005), it has been found that it is important to take contextual factors into account when using feedback in group therapy: not only patient and therapist factors are related to using FIGT, but group therapeutic factors (e.g., cohesion, engagement and avoidance) as well. The results suggest that even when FIGT is more feasible and usable, there may be a risk on focusing too much on group therapeutic factors which can decrease or delay individual therapeutic change, which is less desirable in short-term group therapy. On the other hand, it is possible that long-term therapeutic change could be observed, as group cohesion appears to be an important predictor of group therapy outcome (Burlingame et al., 2018a).

## Coaching Form

Name: *Mary-Ann* Date: *May 25th*                                                                   Coach: *Susan*

Co-therapist: *Barbara*

### Instruction

- Make a print screen of a feedback report from your group with only first names and no patient personal information
- Attach the print screen along with this form in a secured email for the coach

**Table:**

	Yes	No
1. Did you view the feedback report prior to coaching consultation?	X	
2. Did you discuss the feedback report with your co-therapist?		X
3. Did you discuss the feedback report with the group?	X	
4. Did you understand everything of the feedback report?		X

## What Are Your Goals for This Coaching Consultation?

1. I want to know what to do if a patient does not progress for 5 sessions
2. I want to know what I can do if there is no progression on the OQ-45, but there is progress on the personal treatment goals
3. I want to agree with my co-therapist how we can discuss the results of the feedback before each session

## Appendix 2: Structure of the Coaching Session Based on the G.R.O.W. Model

Goal:

- What would you like to cover today? Where would you like to start?
- Where would you like to be by the end of our 45 min?

Reality:

- Do group members fill out the forms on time?
- Is the feedback consistent with your clinical impressions?
- What is this feedback telling you?
- Do you have concerns about sharing and discussing the feedback in the group?

Options and obstacles:

- What questions can we come up with about what we see and what could we learn?
- How might your patients benefit from seeing the feedback?
- How can group members benefit from discussing the feedback together?
- Do the following aspects have impact on how feedback can be used: phase of the group process, norms in the group, interpersonal difficulties?
- What role would you like to have as a group leader and what role would you like the group members to take when using feedback?
- What might get in the way?

Way forward:

- What actions will you take next, and when?
- What are you taking away from our coaching session today?
